# Prevalence, risk factors and health consequences of soil-transmitted helminth infection on the Bijagos Islands, Guinea Bissau: A community-wide cross-sectional study

**DOI:** 10.1371/journal.pntd.0008938

**Published:** 2020-12-16

**Authors:** Olivia Farrant, Tegwen Marlais, Joanna Houghton, Adriana Goncalves, Eunice Teixeira da Silva Cassama, Marito Gomes Cabral, Jose Nakutum, Cristovao Manjuba, Amabelia Rodrigues, David Mabey, Robin Bailey, Anna Last

**Affiliations:** 1 Clinical Research Department, Faculty of Infectious and Tropical Diseases, London School of Hygiene & Tropical Medicine, London, United Kingdom; 2 Region Sanitaria Bolama-Bijagós, Bubaque, Guinea Bissau; 3 Ministry of Public Health, Bissau, Guinea Bissau; 4 Bandim Health Project, Bissau, Guinea Bissau; Emory University, UNITED STATES

## Abstract

Soil-transmitted helminths (STH) are endemic and widespread across Sub-Saharan Africa. A community wide soil-transmitted helminth (STH) prevalence survey was performed on the island of Bubaque in Guinea-Bissau using both Kato-katz microscopy and qPCR methodology. Predictors of infection and morbidity indicators were identified using multivariable logistic regression, and diagnostic methods were compared using k statistics. Among 396 participants, prevalence of STH by microscopy was 23.2%, hookworm was the only species identified by this method and the mean infection intensity was 312 eggs per gram. qPCR analysis revealed an overall prevalence of any STH infection of 47.3%, with the majority *A*. *duodenale* (32.3%), followed by *N*. *americanus* (15.01%) and *S*. *stercoralis* (13.2%). *A*. *lumbricoides*, *and T*. *trichiura* infections were negligible, with a prevalence of 0.25% each. Agreement between diagnostic tests was k = 0.22, interpreted as fair agreement, and infection intensity measured by both methods was only minimally correlated (R_s_ = -0.03). STH infection overall was more common in females and adults aged 31–40. STH infection was associated with open defaecation, low socio-economic status and further distance to a water-source. The prevalence of anaemia (defined as a binary outcome by the WHO standards for age and sex) was 69.1%, and 44.2% of children were malnourished according to WHO child growth standards. Hookworm infection intensity by faecal egg count showed no statistically significant association with age (R_s_ 0.06) but *S*. *Stercoralis* infection intensity by qPCR cycle threshold was higher in pre-school aged children (R_s_ = 0.30, p-value 0.03) There was no statistically significant association between STH infection and anaemia (OR 1.0 p = 0.8), stunting (OR 1.9, p-value 0.5) and wasting (OR 2.0, p-value 0.2) in children. This study reveals a persistent reservoir of STH infection across the community, with high rates of anaemia and malnutrition, despite high-coverage of mebendazole mass-drug administration in pre-school children. This reflects the need for a new strategy to soil-transmitted helminth control, to reduce infections and ultimately eliminate transmission.

## Author summary

To date, the overall aim of the WHO policy of STH control through periodic mass-drug administration (MDA) has been morbidity control through a reduction in infection intensity in school-aged children. The main limitation of this approach is the rapid re-infection rate, both from the environment in areas with poor sanitation, and the persistent reservoir in adults who are not included in the MDA programme. As a consequence of this limitation, there have been calls to change the overall aim from morbidity control to the elimination of STH infection altogether by breaking the transmission cycle through community-wide deworming. However, there is a dearth of epidemiological data pertaining to adults in relation to STH and in addition, most surveys exclude the helminth *Strongyloides stercoralis*, due to limitations of the diagnostic methods used. Our study aims to provide up-to-date epidemiological data of STH prevalence, individual and household risk-factors for infection and the association with anaemia and malnutrition in children under 5 years. From 396 participants who provided a stool sample, we found a high prevalence of STH infection, predominantly the hookworm *Ancylostoma duodenale*, across all age-groups and both sexes. There was a clear association between STH infection and open defaecation in this study, with use of a protected pit latrine protective against infection. There were high rates of anaemia and malnutrition which had no statistically significant association with STH infection, and are likely multifactorial. This study uncovered an ongoing reservoir of STH infection despite high coverage with mebendazole MDA, high rates of morbidity alongside the STH infection and a clear association between STH infection and inadequate sanitation. This reflects the need for further interventional research to assess the optimum strategy to alleviate morbidity and break the STH transmission cycle in this population.

## Introduction

Soil transmitted helminth (STH) infection affects a quarter of the world’s populations, and is considered a neglected tropical disease by the World Health Organization (WHO). This neglect is characterized by a paucity of research, a lack of development of both diagnostics and treatment strategies, and a resultant continuation of the poverty cycle in the poorest communities [1]. The STH consist of five species of nematode: *Ascaris lumbricoides* (roundworm), *Strongyloides stercoralis*; *Trichuris trichiura* (whipworm), and the hookworms; *Necator americanus* and *Ancylostoma duodenale [2]*. The consequences of infection range from gastrointestinal upset to the sequelae of anaemia and malnutrition: thwarting educational achievement, impeding work productivity and impacting mortality [3,4].

The route of transmission for *A*. *lumbricoides* and *T*. *trichiura*, is by oral ingestion of ova, while the hookworms and *S*. *stercoralis* are transmitted by transdermal penetration by filariform larvae [5,6]. This commonly reflects a lack of adequate sanitation and hygiene and shoe-wearing, respectively. The difficulties in diagnosis of *S*. *stercoralis* result in it being frequently excluded from STH mapping studies and its prevalence is dramatically under-reported despite, in addition to the above consequences, sometimes causing the ‘hyper-infection’ syndrome in immunocompromised hosts, which has a high mortality [7]. This is of particular public health concern because of the unique ability of *S*. *stercoralis* to undergo auto-infection in the human host, potentially causing life-long infection and morbidity decades after the initial infection [8].

The consequences of all STH infections have a linear relationship to infection intensity, with greater morbidity resulting from higher worm burdens [9]. The disproportionate impact of high worm burdens on cognitive development and growth in children coupled with the logistical opportunity the school infrastructure provides has meant that school-based deworming with the anti-helminthic drugs albendazole and mebendazole has formed the cornerstone of the global public health intervention against STH infection [10]. The aim of this has been to reduce STH-associated morbidity, rather than interrupt transmission. However, this policy has limitations. Older adults, untouched by school-based deworming, have been shown in previous studies to be a reservoir of infection of both hookworm species and *S*. *stercoralis* [11,12]. With regards to *S*. *stecoralis* in particular, the drugs used in school-based deworming, albendazole and mebendazole, have less or no effect on infection and ivermectin is the treatment of choice [13]. In addition, despite high coverage of school-based deworming, with 565 million people treated [14], re-infection rates are high, impeding efforts to interrupt STH transmission and progress to elimination [15]. This can be explained in part by the association of STH and poor sanitation, with research showing that preventative chemotherapy alone will be unable to eliminate STH and only in areas where access to water and adequate sanitation is high is there a real potential for this [16,17].

As a result of these discussions, there have been recent calls to change the World Health Organization policy to community-wide deworming and for the overall aim to be breaking transmission and moving towards elimination of STH infection [18,19]. Ongoing epidemiological information pertaining to risk factors and the patterns of infection is still required to inform this control effort. There has been copious research into the epidemiological patterns of soil-transmitted helminth infections in pre-school aged and school-aged children, due to the global public health approach. This approach has typically excluded adults and has not included *S*. *stercoralis*. This study seeks to redress this imbalance.

The people of the Bijagos Islands in Guinea Bissau, have little access to healthcare and an unacceptably high under-5 and maternal mortality [20]. Communities on the island live in earth houses with either corrugated iron or thatch roofs, with no mains electricity or running water. Diet is mainly fish and rice with vegetables hard to come by in the rainy season. Occupation is mostly agricultural with the rice crop in the rainy season, cashews in May and June and palm oil the rest of the year. The current public health policy for STH control in Guinea Bissau is biannual mebendazole distribution for children aged 6–59 months, with high coverage documented consistently [21]. A previous study on the Bijagos Islands, published in 1987, found a community prevalence of STH of 80.9% [22], a prevalence of anaemia in children of 80.2% and of underweight children of 9.4% [23]. To date there has been no community-wide study in Guinea Bissau measuring STH including *S*. *Stercoralis*, anaemia and malnutrition. This study addresses this need for research, to estimate the current prevalence of STH, the intensity of infection and the relationship with anaemia and malnutrition, to accurately inform future public health intervention.

## Methods

### Ethics statement

All participants gave informed written consent prior to their involvement, with those under 18 years having written consent given by their parents or guardians in addition to written assent for 7–17 year olds. The research protocol was approved by the Comite Nacional de Etica na Saude in Guinea Bissau and by the London School of Hygiene and Tropical Medicine Ethics Committee.

### Study design

Community-based cross-sectional study in villages on the island of Bubaque, in the Bijagos Archipelago of Guinea Bissau performed in October 2017.

### Study population and study area

The study population was all people living on Bubaque Island, excluding infants under 6 months.

### Sampling methodology

Prior to the study, all buildings, villages, roads, paths and coastlines on all islands of the archipelago were mapped using Open Street Map in collaboration with the Missing Maps Project [24]. Following this, random GPS points were generated using the random point generation tool on QGIS software for each of the fourteen villages on Bubaque. On arrival at each village the team would walk to the randomly selected GPS point and offer participation to all members of the nearest house. Probability proportional to size (PPS) methodology was used to ensure accuracy in sample design as the villages vary in size. The population used for the PPS method was taken from the last count of the 2009 census. A non-proportional sample was taken from the single town on the island, to include samples from the urban environment.

### Sample collection and processing

The Kato-Katz method was used for stool analysis in the field. The stool sample was first pressed through a sieve then collected with a spatula. This was then placed in a 41.7 mg template on top of a microscope slide. After the template was removed, glycerol-soaked cellophane was applied to the slide and then pressed gently to enable faecal material to be spread evenly. All slides were read within 60 minutes to allow identification of hookworm eggs. A positive result was visualisation of any eggs or larvae of the four STH as this technique is not suitable for *Strongyloides*. In addition, an almond-sized amount (approx. 0.5–1 g) of each sample was transferred into a screw cap 2 ml tube with 1 ml of 70% ethanol, stored at ambient temperature and transported to the reference laboratory, at which point samples were frozen at -20°C.

10% of Kato-Katz samples were double-read by an alternative trained microscopist to ensure quality control. If there were differences, the slide was read a third time and a consensus reached, this occurred twice during the study.

### Stool DNA extraction

Stool samples in ethanol were thawed at ambient temperature and homogenized using a FastPrep at 4.0 m/s for 10 seconds. After this, 0.5 ml of each sample was transferred into a clean tube and centrifuged at 9300 xg for 1 minute to pellet the sample. Ethanol supernatant was removed and the pellet was re-suspended in 1 ml phosphate buffered saline (PBS) to wash it. After centrifuging as before, the supernatant was removed and the stool pellet was re-suspended in 800 ul of CD-1 buffer from a DNeasy PowerSoil PRO kit (Qiagen). These samples were heated at 65°C for 10 minutes, then 95°C for 10 minutes in a heat block. The heat-treated samples were then transferred into bead tubes from the kit. Homogenisation/bead beating was optimized for DNA quality and recovery in a FastPrep, with final conditions of 6.0 m/s for 40 seconds, with a 5 minute pause followed by another 40 s of homogenisation. Sample processing was continued from step 3 of the experienced user protocol in the DNA extraction kit and the final elution of purified DNA was in 50μl of elution buffer.

### STH detection by qPCR

*S*. *stercoralis* was analysed as a singleplex and the other 4 species as a multiplex. *S*. *stercoralis* qPCR was as per Verweij et al [25], the separate multiplex was optimized from various methods, as described below. Fluorophores were modified as needed in order to be detected by a Rotorgene 3000. Primer and probe sequences are given in Table 1.

**Table 1:** Primer and probe sequences used in qPCR detection of STH in stool DNA. Bases in brackets and indicated by a + are locked nucleic acids. *S*. *stercoralis* was run separately while the other four species were multiplexed.

Species	Target	Primer	Sequence 5’-3’	Final concentration (nM)	Reference
*Ascaris lumbricoides*	ITS1	Forward	GTAATAGCAGTCGGCGGTTTCTT	60	Basuni 2011
		Reverse	GCCCAACATGCCACCTATTC	60	
		Probe	[FAM]TTGGCGGACAATTGCATGCGAT[BHQ1]	100	
*Ancylostoma*	ITS2	Forward	GAATGACAGCAAACTCGTTGTTG	200	Basuni 2011, Mationg 2017 for LNAs [26]
		Reverse	ATACTAGCCACTGCCGAAACGT	200	
		Probe	[Cy5] ATC[+G]TTTA[+C][+C][+G]A[+C]TTTAG [BHQ2]	200	
*Necator americanus*	ITS2	Forward	CTGTTTGTCGAACGGTACTTGC	200	Basuni 2011, Mationg 2017 for LNAs
		Reverse	ATAACAGCGTGCACATGTTGC	200	
		Probe	[JOE] CT[+G]TA[+C]TA[+C][+G][+C][+A]TT[+G]TATAC [BHQ1]	100	
*Trichuris trichiura*	ITS1	Forward	TCCGAACGGCGGATCA	60	Mejia R et al. 2013 [27]
		Reverse	CTCGAGTGTCACGTCGTCCTT	60	
		Probe	[ROX]TTGGCTCGTAGGTCGTT[BHQ2]	100	
*Strongyloides stercoralis*	18S RNA	Forward	GGGCCGGACACTATAAGGAT	100	Verweij JJ et al. 2009
		Reverse	TGCCTCTGGATATTGCTCAGT	100	
		Probe	[FAM]ACACACCGGCCGTCGCTGC[BHQ1]	100	

The PCR mix used Probe Mix No-Rox (PB20.23, PCR Biosystems) and consisted of final concentrations of forward primer, reverse primer and probe as stated in Table 1. Reactions used 3 μl of sample DNA in a total volume of 25μl for both the *S*. *stercorlais* singleplex and the multiplex. In addition, the multiplex contained bovine serum albumin to a final concentration of 0.1 mg/ml to increase the efficiency of *N*. *americanus* target amplification.

Thermal cycling conditions were based on the method by Basuni et al 2011 [28] but optimized on a Rotorgene 3000 and were, for *S*. *stercoralis*: 95°C for 10 secs, then 50 cycles of 60°C for 1 minute. For the multiplex, conditions were: 95°C for 2 minutes, followed by 5 cycles of 95°C for 9 s, 64–60°C (touchdown) for 1 minute, then 50 cycles of 95°C for 9 s and 60°C for 1 minute, during which data was acquired on the FAM, Cy5, JOE and ROX channels, corresponding to the fluorophores used. A target-DNA-free negative control and positive control DNA for each species were included in each run.

Positive control DNA originated from larvae of *S*. *stercoralis*, plasmids containing synthetic GeneArt Strings (Invitrogen by ThermoFisherScientific) of the target sequence of *A*. *duodenale* and cloned target sequences from *N*. *americanus*, *A*. *lumbricoides* and *T*. *trichiura* positive stool DNA. Samples and controls were classed as positive if they crossed the fluorescence threshold at or before cycle 40. The threshold was set in the exponential part of the amplification curve, above background level, and was consistent between runs.

Haemoglobin measurements were taken from all participants using a HemoCue Hb 201^+^ analyser with fingerprick blood samples. For the nutritional survey, height of children under 5 years was measured using a tape measure standing against a wall in centimetres, and weight using a standard electronic scale.

Individuals were asked for basic socio-demographic information and in addition, heads of household were asked to complete a survey pertaining to sanitation and hygiene practices. This included questions regarding main water source, distance to water source, access to sanitation, hygiene practices and shoe-wearing habits. Information regarding access and take-up of mebendazole MDA was also asked in relation to children under 4 years. Direct observation was used to validate presence of soap and the type of water source.

### Outcomes

The primary outcome was STH prevalence. Secondary outcomes were risk factors for STH and morbidity associated with STH, reflected as presence of anaemia and malnutrition in children under 5 years.

### Statistical analysis

Sample size calculation was estimated using an estimated prevalence of 56%, a design effect of 2.5 (23), a confidence level of 95% and an estimated population of 2744 from GIS population estimation. This gave a sample size of 383 people at 80% power.

Descriptive analysis was performed using Stata 16 to examine socio-demographic information; STH prevalence and infection intensity by microscopy and qPCR; water, sanitation and hygiene (WASH) access and use; anthropometric indices; and anaemia. Data from qPCR analysis was used when exploring associations with STH infection as a categorical variable. Data from microscopy in the form of infection intensity as eggs per gram was used as a continuous variable, and as cycle threshold when using qPCR data. Presence of anaemia was used as a categorical variable using the WHO groupings by age-group and sex [29], and haemoglobin as a continuous variable. Anthropometric data was analysed in Stata 16 using the WHO Anthro package to calculate relevant z-scores with standard deviations and confidence intervals [30]. Weight, height, height-for-age (HAZ), weight-for-age (WAZ) and weight-for-height (WHZ) was calculated for each participant child. Children were classified as stunted if HAZ <-2 SD from the mean, underweight if WAZ <-2 SD from the mean and wasted if WHZ <-2 SD from the mean [31]. Values were excluded from the analysis if they were implausible according to the WHO Anthro package. Ordinal logistic regression was used in the analysis of categorical outcomes, Pearsons or Spearman’s correlation were used to analyse continuous data, depending on distribution. The agreement between the diagnostic methods was assessed using Cohen’s kappa. The k-statistic was interpreted as < 0.00, poor agreement; 0.00–0.20, slight agreement; 0.21–0.40, fair agreement; 0.41–0.60, moderate agreement; 0.61–0.80, substantial agreement; 0.81–1.00, almost perfect agreement [32].

## Results

A total of 419 participants were enrolled in this study on Bubaque Island, 396 provided stool samples, 405 had a haemoglobin measurement, 373 completed the WASH questionnaire and 79 children completed the anthropometric assessment.

### Baseline assessment

Participants were mostly adults (222/405, 54.8%) and female (229/405, 56.5%). The majority of participants reported ownership of an electrical item (60.3%), thus conferring high socioeconomic status as measured in this study. 89.2% (333/373) of participants reported use of an improved water source and 76.08% (283/373) of participants reported the time taken to walk to their principal water source, collect water and return home was less than 30 minutes (mean 25.1 ±14.7). 64.3% (240/373) of participants reported no access to any sanitation facility and practiced open defecation. Soap was observed as present in 22.2% (83/373) of participant’s homes.

### STH prevalence

Prevalence of STH by microscopy was 23.2% and hookworm was the only species identified by this method. qPCR analysis revealed an overall prevalence of any STH infection of 47.3%, see Table 2. *A*. *duodenale* (32.3%) and *N*. *americanus* (15.01%) were the most prevalent species identified, followed by *S*. *stercoralis* (13.2%). *A*. *lumbricoides*, *and T*. *trichiura* were found in only one participant each, with a prevalence of 0.25% respectively. 44 participants (11.1%) had a mixed infection with two or more species of helminth identified by qPCR. STH infection displayed a highly heterogenous distribution seen across villages with a range in prevalence from 22.4% in Bruce to 77.7% in Tcharo. The overall mean infection intensity of participants with hookworm eggs demonstrated by microscopy was 312 eggs per gram of faeces (95% CI 161.0–463.0), with the majority light infections as described by the WHO (Eggs per gram—EPG <1999). There was a range from 24–5904 EPG with one participant showing a moderate intensity infection (2784 EPG) and one showing a heavy intensity infection (5904 EPG).

**Table 2:** STH prevalence by microscopy and PCR.

	Number/Total (%)
Hookworm infection* **by KatoKatz microscopy**	
Yes	92/396 (23.23)
**STH infection by qPCR (%)**	
Any STH	186/393 (47.3)
*A*. *duodenale*	127/393 (32.3)
*N*. *americanus*	59/393 (15.01)
*S*. *stercoralis*	52/393 (13.2)
*A*. *lumbricoides*	1/393 (0.25)
*T*. *trichiura*	1/393 (0.25)

*No other species was identified by microscopy.

### Risk factors for STH infection

**Fig 1:**
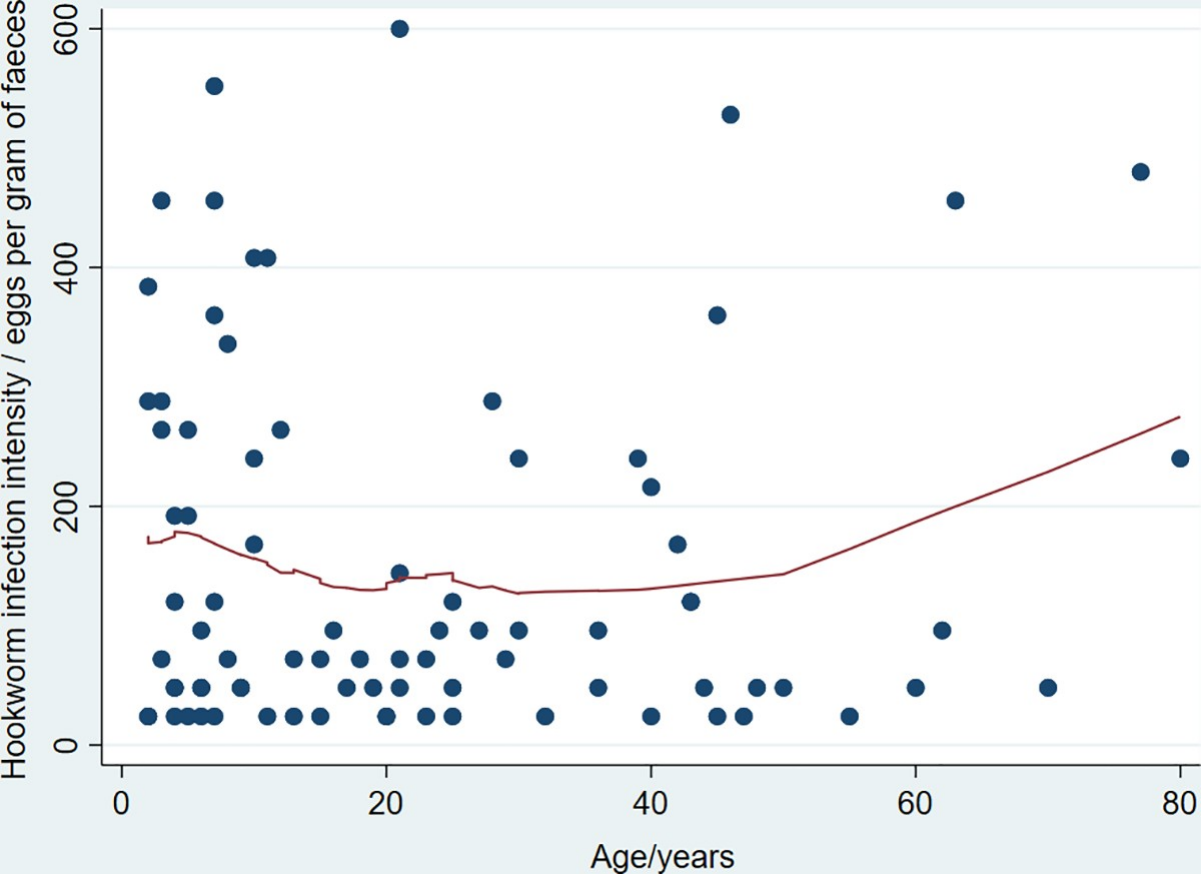
Lowess demonstrating hookworm infection intensity by age.

Hookworm infection intensity measured by faecal egg count was not significantly correlated with age (R_s_ = 0.06, 95% CI -0.15 to 0.27, p = 0.56) see Fig 1. *S*. *stercoralis* infection intensity using cycle threshold level from qPCR data was positively correlated with age; R_s_ = 0.30 (95% CI 0.01 to 0.53, p-value 0.03) denoting a decrease in infection intensity with increasing age. *S*. *stercoralis* was predominantly an infection of pre-school aged children (OR 0.30, p = <0.01) with a prevalence of 23.4% in this age group.

**Fig 2:**
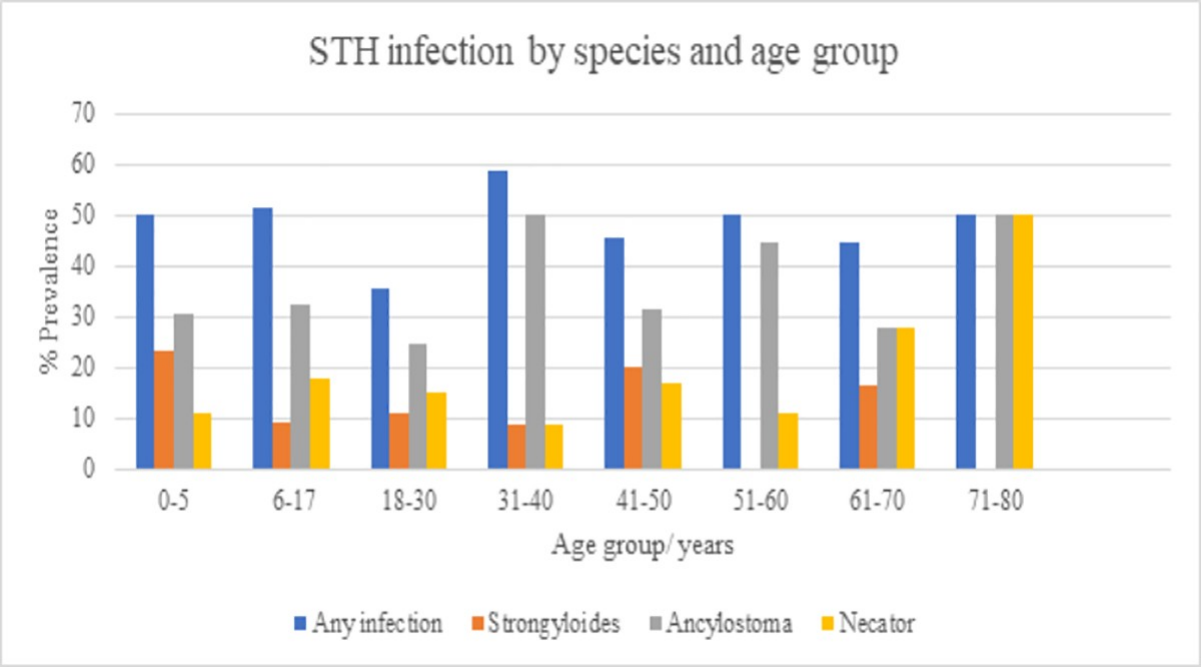
Prevalence of STH species by qPCR by age group.

STH infection was seen most commonly in school-aged children, OR 1.10 p = 0.74, though this was not statistically significant. The overall trend across species was a high prevalence in the 0–5 age group (50%), sustained across childhood and adolescence (51.3%), a drop in prevalence in young adults aged 18–30 (35.6%) and prevalence consistently over 40% in adults aged over 40, see Fig 2. STH infection did not differ significantly between the sexes overall across age groups (OR 1.19, p = 0.40) but in adults it was seen more commonly in women (OR 1.29, p = 0.42), though this was not statistically significant. When age was further subcategorized, this was most of note in the 18–30 age group with women more likely to be infected, OR 2.06, p = 0.19 but this was not statistically significant.

There was weak evidence to suggest ownership of an electrical item (OR 0.75, p = 0.19), and reduced distance to a water source (OR 0.80, p = 0.39) were associated with reduced STH infection. There was stronger evidence to suggest use of a protected pit latrine (OR 0.62, p = 0.03) and living in an urban environment (OR 0.31, p = <0.01) were protective against STH infection. Multivariate analysis of STH infection and protected pit latrine access retained statistical significance when adjusted for age, sex, socio-economic status, water-source and distance to water-source (AOR 0.74, 95% CI 0.58–0.94, p 0.01).

### Anaemia

The overall prevalence of anaemia as a binary outcome using the WHO standards by age and sex was 69.1% across all demographics. The highest prevalence was seen in adolescents age 12–14 with a prevalence of 82.6%. Adult females were more likely to be anaemic than adult males, OR = 1.63, p = 0.08, but this was not statistically significant. Severe anaemia, defined as a haemoglobin <70g/l in pre-school aged children and <80g/l in other demographic groups, was seen in 11 participants (2.7% of the study population).

There was no significant association between overall STH infection by qPCR and anaemia (OR 1.03, p-value 0.87). Anaemia was not significantly associated with *S*. *stercoralis* infection (OR 1.72, p-value 0.13), *A*. *duodenale* (OR 0.88, p-value 0.60) or *N*. *americanus* infection (0.98, p-value 0.95). A Spearman’s Rank Correlation was performed to assess the relationship between haemoglobin as a continuous variable and intensity of hookworm infection by faecal egg count. A linear negative correlation was found but this was not statistically significant. The Spearman’s correlation coefficient, R_s_ was negative: -0.16 (p = 0.11).

Children who were underweight and children who were wasted were more likely to be anaemic (OR 1.84, p-value 0.36 and OR 1.28, p-value 0.72), but neither of these differences were statistically significant.

### Anthropometric survey

The anthropometric survey highlighted 31/70 children under 5 who were malnourished using any indicator (HAZ, WHZ and WAZ), giving an overall prevalence of 44.2%. Stunting was observed at a prevalence of 4.2%, with a mean height for age score (HAZ) of 1.02 (SD± 3.15). Wasting was measured at a prevalence of 41.0% with a mean height for weight score (WHZ) of -2.16 (SD± 3.43). 38.9% of children under 5 were underweight, with a mean weight for age (WAZ) score of -0.38 (SD± 3.81) (Table 3). Children with STH infection by qPCR with more likely to have stunting (OR 1.9, p-value 0.5) and wasting (OR 2.0, p-value 0.26), but neither of these differences were statistically significant.

**Table 3:** Baseline characteristics of study population.

Characteristic	Number/Total (%)	Infected any STH by PCR/total N (%)	OR	95% CI	p-value
**Age group**					
Pre-school aged children (0–4)	85/405 (20.9)	40/85 (47.0)	1		
School-aged children (5–17)	120/405 (29.6)	58/120 (48.3)	1.10	0.62–1.95	0.74
Adults (>18)	200/405 (49.4)	80/200 (40.0)	0.81	0.48–1.37	0.43
**Sex**					
Male	176/405 (43.4)	75/166 (45.1)	1		
Female	229/405 (56.5)	103/208 (49.5)	1.19	0.78–1.79	0.40
**Electrical item ownership**					
No	148/373 (39.6)	74/143 (51.7)	1		
Yes	225/373 (60.3)	98/219 (44.7)	0.75	0.49–1.15	0.19
**Drinking water-source**					
Unimproved	40/373 (10.7)	12/36 (33.3)	1		
Improved	333/373 (89.0)	160/326 (49.0)	1.92	0.93–3.98	0.07
**Distance water source**					
>30mins	90/373 (24.1)	36/83 (43.3)	1		
<30mins	283/373 (76.08)	136/279 (48.7)	0.80	0.49–1.31	0.39
**Sanitation**					
Unimproved	240/373 (64.3)	120/232 (51.7)	1		
Improved	133/373 (35.6)	52/130 (40.0)	0.78	0.63–0.98	0.03
**Hygiene**					
Soap not observed	290/373 (77.7)	124/278 (44.6)	1		
Soap observed	83/373 (22.2)	48/83 (57.8)	1.70	1.03–2.79	0.03
**Environment**					
Rural	356/419 (84.8)	170/332 (51.2)	1		
Urban	63/419 (15.0)	14/56 (25.0)	0.31	0.16–0.60	0.00
**Anaemia**					
No	125/405 (30.8)	55/117 (47.0)	1		
Yes	280/405 (69.1)	123/257 (47.8)	1.0	0.6–1.6	0.8
**Children nutritional status**					
**Stunting**					
No	69/72 (96.0)	33/65 (50.7)	1		
Yes	3/72 (4.2)	2/3	1.9	0.1–22.4	0.5
Underweight					
No	33/54 (61.1)	16/30 (53.3)	1		
Yes	21/54 (38.9)	9/21 (42.8)	0.6	0.21–2.0	0.4
Wasting					
No	26/44 (59.0)	10/23 (43.4)	1		
Yes	18/44 (41.0)	11/18 (61.1)	2.0	0.58–7.17	0.6

Note: 373 participants completed the WASH questionnaire, 405 had a haemoglobin measurement and 393 had qPCR performed on a stool sample. The denominator in the second column of each category differs from the numerator of the first column because not all participants were able to provide a stool sample.

### Comparison of diagnostic tests

**Fig 3:**
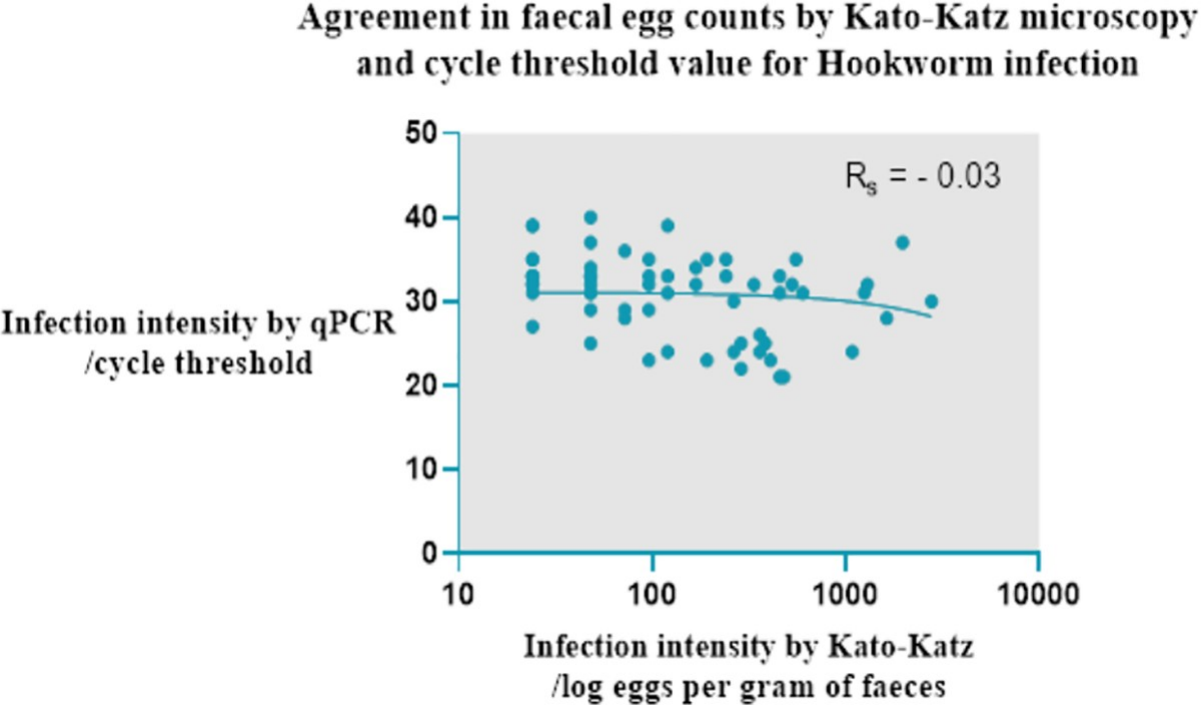
Agreement in infection intensity by qPCR and Kato-Katz microscopy.

The agreement between tests in this study was k = 0.22 from the data in Table 4, interpreted as fair agreement. Spearman’s correlation of infection intensity by qPCR cycle threshold and faecal egg count by Kato-Katz was R_s_ = -0.03 (95% CI -0.24–0.17, p-value 0.7), shown in Fig 3. There were 27 samples in which hookworm eggs were found by Kato-katz microscopy but were subsequently PCR negative, see Table 4. Similarly, 122 samples that were negative by Kato-katz microscopy were then found to be PCR positive.

**Table 4:** 2x2 table of PCR and Kato-katz results.

	Positive by Kato-katz method		
Positive by PCR	Yes	No	Total
Yes	64	122*	186
No	27	177	204
	91	299	390

*Including the 2 samples which tested positive for *A*. *Lumbricoides* and *T*. *Trichuiris*.

## Discussion

The WHO defines hookworm infection to be of public health concern when the prevalence of moderate and high intensity infections is greater than 1% in school-aged children [33]. In our study the majority of participants had light infections, which is in line with the observed epidemiological patterns of STH infection in endemic settings, with a few individuals harbouring the majority of adult worms [34]. Previous research on the Bijagos Islands estimated a prevalence of *A*. *duodenale* of up to 87.9% [22], higher than our estimate of 32.3%. This may be explained by access to mebendazole mass drug administration among pre-school aged children, or possibly improved socio-economic status of the population since the last study reflected in the ubiquitous livestock ownership and high proportion of electrical ownership in this study. In addition to this, although the Ebola epidemic did not reach Guinea-Bissau in 2015, there was a significant public health effort to raise awareness of the importance of hand washing and strategies to limit the spread of infection. In contrast to this, *S*. *stercoralis* not only showed similar prevalence estimates to previous research, but also was found predominantly in pre-school aged children, the demographic targeted with mebendazole mass drug administration. It is globally accepted that at a prevalence of greater than 5% it is considered hyper-endemic [35] and this study shows both that this has not changed despite the possible improvements in socio-economic status and that mebendazole has no impact on *S*. *stercoralis* endemicity.

The most prevalent STH species was *A*. *duodenale*, followed by *N*. *americanus* and *S*. *stercoralis*. All participants reported regular shoe wearing, which may explain the higher rates of *A*. *duodenale* over *N*. *americanus* and *S*. *stercoralis*. Although not commonly recognised, the larvae of *A*. *duodenale* can be transmitted by oral ingestion, in addition to transdermal penetration, hence its reputation as the more pathogenic organism [36]. In certain endemic settings it has been shown the oral route of transmission of *A*. *duodenale* predominates which may explain the higher prevalence in the study area [37]. Only two participants were infected with *A*. *lumbricoides* or *T*. *trichiura* in this study, despite previous prevalence estimates of 38.4% of *T*. *Trichiuria*, compared to *A*. *lumbricoides* which has always been found at low prevalence in this study area [22]. This is a surprising finding, given mebendazole MDA has been shown to be ineffective in many cases of Trichuriasis so this would not be expected to explain this reduction in infection prevalence [38].

STH infection was most common in adults aged 31–40 years, in line with previous research demonstrating *A*. *duodenale*, the predominant species in our study, to be more common in adults [11]. It is also notable that the lowest prevalence of STH infection was seen in the 18–30 age-group, and within this, females were more likely to be infected than males (OR 2.06, p = 0.19). This may be because in the Bijagos Islands, unlike many in Sub-saharan Africa, communities have a matriarchal structure, with women typically working in farming more than men, a known risk factor for hookworm infection [39, 40]. The age-intensity profile of hookworm in this study was linearly positive, reflecting the rising infection intensity into and throughout adulthood.

Kato-katz is the most widely used diagnostic method in epidemiological surveys due to its ease of use and low-cost. Microscopy using the Kato-katz method in our study demonstrated a prevalence of 23.23%, compared to a qPCR prevalence of 47.3%, with an agreement between tests of k = 0.22. This is in comparison to other studies measuring hookworm and *S*. *stercoralis* which found an agreement of k = 0.63 between PCR and Kato-katz [7]. The limitations of Kato-katz are predominantly associated with the low sensitivity at lower infection intensity [41], as in our study, which may explain this large difference in microscopy prevalence compared to qPCR. In a recent study in the capital of Guinea-Bissau that used solely Kato-katz microscopy, the prevalence of hookworm was found to be only 5.9%, with a similar risk factor profile as our study population, which may reflect the limitations of the diagnostic method [42]. In our study, there were 27 samples that were positive for hookworm infection by Kato-katz microscopy but subsequently PCR negative. This may reflect a combination of factors including PCR inhibitors in stool, and human error in the identification of helminth ova by microscopy. Such discrepancy has been found by other studies, even in comparison with triplicate Kato-katz [26]. We optimised the conditions of the multiplex qPCR assay for the STH using positive control material, however we did not use an extraction and amplification control, which this assay could benefit from to ensure effective DNA extraction and lack of inhibition. When comparing qPCR and Kato-katz hookworm infection intensity, there was weak evidence to suggest a correlation. This is important to recognize as the sensitivity of diagnostic tests to diagnosis helminth infections will either enable or hinder any public health campaign’s ability to monitor its progress, assess the impact of interventions and decide when to stop the programme. It may be that as infection intensity reduces due to successful MDA programmes, other diagnostic tests that are more sensitive at lower infection densities, such as qPCR, would be more suitable.

This was the first community-based survey of anaemia on the Bijagos and revealed extremely high rates across all age groups, such to warrant significant public health concern [43]. The group with the highest prevalence of anaemia was adolescents aged 12–14, followed by pre-school aged children and school-aged children. Anaemia was more common in adult females than adult males (OR 1.63, p = 0.09.) In our study there was no strong association between anaemia and STH infection overall. This supports evidence from other studies that suggest an underlying multifactorial causality of anaemia, with malaria, HIV, bacteremia, G6PD deficiency, Vitamin A and Vitamin B12 deficiency playing a role, in comparison to other studies in Sub-saharan Africa [44]. Malarial anaemia is likely to be the principal cause in our study, given that it took place in the rainy season [45]. Adult females had significantly higher rates of anaemia than adult males in this study, which is likely to be related to maternal anaemia. Of note, this has been shown to have a positive linear relationship with childhood anaemia: the lower the haemoglobin of the mother, the higher the odds of anaemia in the child, which may explain in part the high rates of anaemia in pre-school aged children in this study [46].

This study highlighted high rates of malnutrition in children under 5 with an overall prevalence of 44.2%. The most prevalent indices of malnutrition were wasting and underweight, which are typically measures of acute malnutrition [47]. Chronic malnutrition or stunting, represented in the measurement of height-for-age was not common, at a prevalence of 4.2%. This is in contrast to the nation-wide Multiple Indicator Cluster surveys in 2006 which showed a prevalence of stunting of 48% [48]. It is encouraging that this underlying burden of chronic malnutrition has lessened, however it seems the more acute markers of malnutrition, represented in wasting and thinness/underweight have increased as seen in our study. This may be explained in part by STH infection, which was more likely in children with wasting (OR 2.0, p = 0.2) despite weak evidence. In addition, both underweight and wasted children were more likely to be anaemic (OR 1.84, p = 0.3 and OR 1.28, p = 0.7). It may also be explained by the seasonal nature of food availability during rainy season when this study took place, which has been shown to be inadequate previously in the same study area [23].

This study highlights the lack of sanitation on Bubaque Island, with the majority of participants practicing open defaecation. There was strong evidence to suggest use of a protected pit latrine was a protective factor against infection with STH compared to open defaecation, retaining strength of association when adjusted for confounding (AOR 0.74, p 0.01). The beneficial effects of protected latrine use on STH infection is well documented but this is more notable with *A*. *lumbricoides* and *T*. *trichiura*, with shoe-wearing having a greater impact on hookworm infection [16]. In our study, shoe-wearing was reported as ubiquitous, but this was self-reported, not observed. Handwashing, our measure of hygiene in the study, was reported by 98.8% of participants but soap was observed in the households of 22.2% of participants. This reflects the universal knowledge of the importance of this practice among even the most isolated of communities but the well-known phenomenon of adults over-reporting ‘desirable’ behaviours to interviewers [49]. The mean time to a water source in this study was 25.1 minutes, and the principal water source used was a protected spring, which attains the minimum standards for access to water by the Sustainable Development Goals and reflects a lower risk of infection with water-borne pathogens [50]. The link between adequate access to water, sanitation and hygiene practices and STH infection has been made on an observational basis but to date there has been no clear method of sanitation intervention that has been shown to successfully reduce the prevalence of STH infection [51,52], likely due to sub-optimal latrine use in the intervention arms. Attempts at both community-led total sanitation and the provision of subsidies to those using latrines have been implemented in some areas with some success in increasing use [53]. In addition to STH infection and other gastrointestinal parasites, latrine use has also been shown to have an impact on child growth indicators, with a reduction in stunting seen in communities provided with latrines [54]. No such sanitation interventions have been attempted on the Bijagos Islands despite the beneficial impact seen in this study.

The limitations of this study are typical of working in a low-income setting. The study was performed during rice-picking season, when most residents of the rural villages are in the fields most of the day. Two of the larger villages in the study move the entire village to a temporary settlement during the season. It may be that rates of open defecation decrease out of rice-picking season, as there are more protected pit latrines in the permanent villages. This study was carried out on only one island in the archipelago and it is that which is best connected to the mainland by boat. The high prevalence of STH seen in the only previous STH epidemiological survey was from more isolated islands which may see higher rates of STH. The anthropometric indices in this study must be considered within the context of generally poor approximation of age of young children and adults. There was no validation done of age as it is rare in this population for there to be any formal identity documentation, including birth certification. In addition, the observational components of the study were not extended to direct observation of shoe-wearing or the use of the soap, rather than soap simply being present.

In summary, there are consistently high rates of infection with hookworm species and *S*. *stercoralis* in the Bijagos Islands despite high coverage of pre-school aged children with biannual mebendazole MDA. The risk factor most strongly associated with STH infection was open defaecation, with high rates demonstrated in the rural areas predominantly. There were also high rates of anaemia and acute malnutrition in the study population, the causes for which are likely multifactorial. If we are to progress towards elimination of STH infection through the interruption of transmission, collaborative approaches with WASH initiatives and mass drug administration with a chemotherapy that includes *S*. *stercoralis* in its therapeutic target are needed to reduce the burden of STH infection in this population. In addition, further research is needed to investigate the causes of the high rates of anaemia and malnutrition identified in this study, in order to develop a sustainable public health approach to improve morbidity and mortality in this community.
